# Generation of Healthy Mice from Gene-Corrected Disease-Specific Induced Pluripotent Stem Cells

**DOI:** 10.1371/journal.pbio.1001099

**Published:** 2011-07-12

**Authors:** Guangming Wu, Na Liu, Ina Rittelmeyer, Amar Deep Sharma, Malte Sgodda, Holm Zaehres, Martina Bleidißel, Boris Greber, Luca Gentile, Dong Wook Han, Cornelia Rudolph, Doris Steinemann, Axel Schambach, Michael Ott, Hans R. Schöler, Tobias Cantz

**Affiliations:** 1 Max-Planck-Institute for Molecular Biomedicine, Münster, Germany; 2 Junior Research Group Stem Cell Biology, Cluster of Excellence REBIRTH, Hannover Medical School, Hannover, Germany; 3 Department of Gastroenterology, Hepatology and Endocrinology, Hannover Medical School, and TWINCORE Centre for Experimental and Clinical Infection Research, Hannover, Germany; 4 Department of Stem Cell Biology, Konkuk University, Seoul, Republic of Korea; 5 Junior Research Group Genetic & Epigenetic Integrity, Cluster of Excellence REBIRTH, Institute of Cell and Molecular Pathology, Hannover Medical School, Hannover, Germany; 6 Junior Research Group Hematopoietic Cell Therapy, Cluster of Excellence REBIRTH, Department Experimental Hematology, Hannover Medical School, Hannover, Germany; 7 Medical Faculty, University of Münster, Münster, Germany; Baylor College of Medicine, United States of America

## Abstract

Using the murine model of tyrosinemia type 1 (fumarylacetoacetate hydrolase [FAH] deficiency; *FAH* ^−/−^ mice) as a paradigm for orphan disorders, such as hereditary metabolic liver diseases, we evaluated fibroblast-derived *FAH* ^−/−^-induced pluripotent stem cells (iPS cells) as targets for gene correction in combination with the tetraploid embryo complementation method. First, after characterizing the *FAH* ^−/−^ iPS cell lines, we aggregated *FAH* ^−/−^-iPS cells with tetraploid embryos and obtained entirely *FAH* ^−/−^-iPS cell–derived mice that were viable and exhibited the phenotype of the founding *FAH* ^−/−^ mice. Then, we transduced *FAH* cDNA into the *FAH* ^−/−^-iPS cells using a third-generation lentiviral vector to generate gene-corrected iPS cells. We could not detect any chromosomal alterations in these cells by high-resolution array CGH analysis, and after their aggregation with tetraploid embryos, we obtained fully iPS cell–derived healthy mice with an astonishing high efficiency for full-term development of up to 63.3%. The gene correction was validated functionally by the long-term survival and expansion of FAH-positive cells of these mice after withdrawal of the rescuing drug NTBC (2-(2-nitro-4-fluoromethylbenzoyl)-1,3-cyclohexanedione). Furthermore, our results demonstrate that both a liver-specific promoter (transthyretin, *TTR*)-driven FAH transgene and a strong viral promoter (from spleen focus-forming virus, *SFFV*)-driven FAH transgene rescued the FAH-deficiency phenotypes in the mice derived from the respective gene-corrected iPS cells. In conclusion, our data demonstrate that a lentiviral gene repair strategy does not abrogate the full pluripotent potential of fibroblast-derived iPS cells, and genetic manipulation of iPS cells in combination with tetraploid embryo aggregation provides a practical and rapid approach to evaluate the efficacy of gene correction of human diseases in mouse models.

## Author Summary

Pluripotent stem cells have unlimited self-renewing capability and the potential to differentiate into virtually all cell types of the body. Pluripotent stem cells are therefore of great interest for future cell-based therapies and are already in use today for studying diseases “in a dish” and screening for new drugs. After the seminal discovery that induced pluripotent stem cells (iPS cells) can be generated by the delivery of four transcription factors into non-pluripotent cells, a tremendous amount of enthusiasm arose about the idea that patient-derived pluripotent stem cells could be obtained and genetically corrected in order to develop customized therapies for regenerative medicine. Here, we present a mouse model of acute metabolic liver failure that fulfills such criteria. First, we demonstrated by stringent assays that disease-specific iPS cells exhibited full cellular and developmental potential and the iPS cell–derived mice reproduced the phenotypes of the founding *FAH* ^−/−^ mice faithfully. Then, we genetically repaired the disease-specific iPS cells by lentiviral delivery of an intact gene copy, and we investigated the impact of this additional genetic manipulation on these cells. With our analyses, we ruled out major, and even minor, chromosomal aberrations in the gene-corrected iPS cells. Most importantly, we demonstrated that the gene-corrected cells maintained their full potential and we generated viable mice that were completely derived from these repaired cells via tetraploid complementation approach, and these mice were healthy, without any signs of the metabolic liver disease.

## Introduction

Direct reprogramming of somatic cells into induced pluripotent stem (iPS) cells has been achieved by the expression of the four factors Oct4, Sox2, Klf4, and c-Myc (or of Oct4, Sox2, Nanog, and Lin28) in mice and humans [1]–[4]. This new technology offers a promising approach for the derivation of disease-specific iPS cells for basic and regenerative medicine research. The technique was first successfully used in a murine sickle cell anemia model [5], demonstrating that gene-corrected iPS cell–derived hematopoietic progenitor cells ameliorated, but did not fully normalize, the phenotype associated with this chronic red blood cell disorder. Various human disease-specific iPS cell lines have been generated [6] either for disease modeling [7]–[16] or for cell transplantation in preclinical models [17]–[20]. However, to date, no report has demonstrated that a full pluripotent phenotype is maintained in gene-corrected iPS cells, which is of particular importance considering the controversy regarding the slightly different differentiation capabilities of iPS and ES cells [21],[22] and the genetic or epigenetic stability of human iPS cells [23]–[26]. These studies have found numerous molecular changes in human iPS cells: chromosomal abnormalities (increased copy number alterations, CNAs), multiple point mutations (partly acquired during the early phase of reprogramming), and aberrant methylation of CG dinucleotides (retained epigenetic marks). However, no correlation between such (epi)genetic alterations and different reprogramming methods has been found, and it remains fairly unclear how those changes affect the functionality of pluripotent stem cells. Therefore, more robust assays to better assess the pluripotency of human and murine iPS cells are needed.

The most stringent test for pluripotency is tetraploid complementation, which uses tetraploid (4n) embryos as host embryos for injection or aggregation with ES cells, resulting in offspring that are entirely derived of ES cells in a single step [27]–[29]. We therefore consider the tetraploid complementation as the state-of-the-art technique to assess the pluripotency of a given cell line, as an organism that has been fully derived from only the cells in question will exhibit their full potential. Wernig et al. utilized this approach to analyze iPS cell–derived fetuses of embryonic day (E) 12.5 [3], but they could not obtain live-born pups. More recently, three groups reported the generation of mice from 4-factor reprogrammed non-diseased iPS cells to adulthood [30]–[32]. Most recently, Stadtfeld et al. used tetraploid embryo complementation to demonstrate that aberrant silencing of the *Dlk1-Dio* cluster on chromosome 12 is the distinguishing characteristic of murine iPS cells capable of contributing to full-term development, versus those incapable [33]. But it is not known whether genetic manipulation and prolonged propagation of iPS cells impairs their potential to support full-term embryo development.

In this study, we derived iPS cells from the fibroblasts of FAH^−/−^ mice, a mouse model of the acute and life-threatening disease tyrosinemia type 1 (fumarylacetoacetate hydrolase [FAH] deficiency) in humans [34], which we considered as a paradigm for an iPS cell–based gene therapy approach for orphan diseases, such as severe metabolic liver disorders. We did not consider gene correction by homologous recombination as the preferred option, as the classic approach of electroporation to introduce BACs and deliver more than 100 kbp of gene flanking regions is accompanied by strong DNA damage (double-strand breaks) and by offsite integrations of part of the transferred DNA [35],[36]. In addition, this approach is rather inefficient and cannot be applied to human ES or iPS cells. The use of tailored zinc fingers attached to an endonuclease for site-specific double-strand breaks substantially increases the efficiency of recombination, but the flanking regions of the repair constructs are only 0.5–1 kbp long, and therefore specific zinc fingers need to be designed for each targeted locus [37]. As the human FAH gene contains 14 exons and is 35 kb long, a single zinc finger–based homologous recombination approach cannot be used to correct the different mutations in newborns with tyrosinemia type I, unless new targeting strategies are designed for each new mutation. In the present study, we generated fully pluripotent iPS cells from the diseased mice and established a gene-corrected iPS cell line by transduction of an intact *FAH* cDNA sequence on a third-generation self-inactivating lentiviral vector, which is much safer in terms of oncogenic transformation than a conventional gamma-retroviral vector [38]. This is because conventional retroviral vectors preferentially integrate next to transcriptional start sites and regulatory gene regions, whereas lentiviral vectors tend to insert far away from the transcriptional regulating sites of transcribed genes [39]. In fact, lentiviral vectors have already been used in the clinical setting as part of the therapeutic intervention for a variety of disease states, including advanced forms of HIV infection, inherited disorders affecting hematopoietic cells, and Parkinson's disease (reviewed in [40]). The same vector architecture as in our experiments was used in a recent gene therapy trial for X-linked adrenoleukodystrophy [41] and is also currently used in an ongoing multicenter trial for Wiskott-Aldrich syndrome, coordinated by the San Raffaele Telethon Institute for Gene Therapy (Milan, Italy; meeting abstract [42]).

To date, no iPS cell–derived differentiation protocol has succeeded in yielding transplantable cells that fulfill both criteria—functional engraftment and response to proliferative stimuli in the diseased liver—and the best evidence for functional hepatic iPS cell–derivatives was recently demonstrated using wild-type iPS cells to supplement *FAH* ^−/−^ blastocysts in chimeric mice [43]. Therefore, we performed tetraploid embryo complementation experiments to demonstrate that gene correction did not affect the pluripotent state of the iPS cells and, most importantly, that gene-corrected iPS cells can be used to generate functional hepatocytes in vivo that fully restore the diseased phenotype of acute liver failure and can be stimulated to proliferate and regenerate liver tissue.

## Results

### Generation and Characterization of *FAH* ^−/−^-iPS Cells

**Figure 1:**
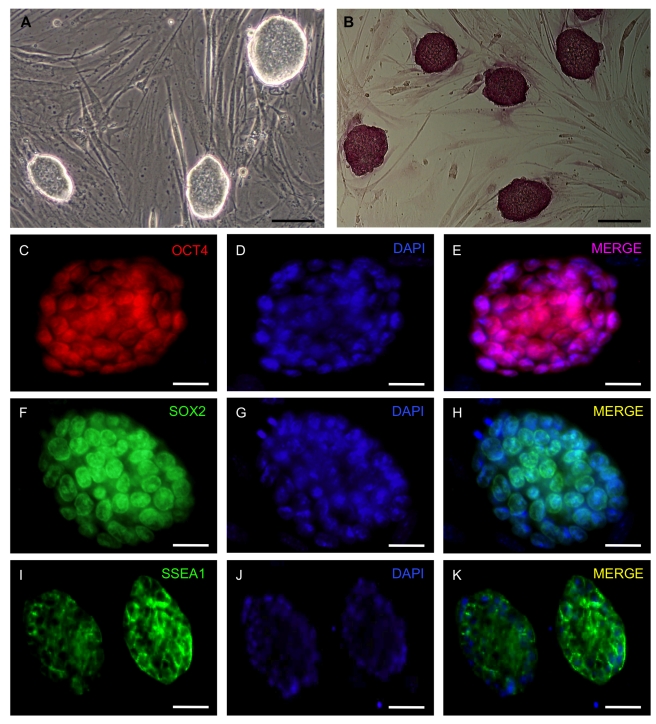
Characterization of iPS cells from *FAH* ^−/−^ mice. (A) Morphology of *FAH* ^−/−^-iPS cells (line #7) grown on MEF cells under murine embryonic stem cell culture conditions. (B) Alkaline phosphatase activity demonstrates homogenous staining in *FAH* ^−/−^-iPS cell colonies. (C–K) Immunofluorescence analyses for Oct4, Sox2, and SSEA1 confirms the induction of pluripotency in *FAH* ^−/−^-iPS cells. Scale bars, 100 µm in (A) and (B) and 50 µm in (C–K).

**Figure 2:**
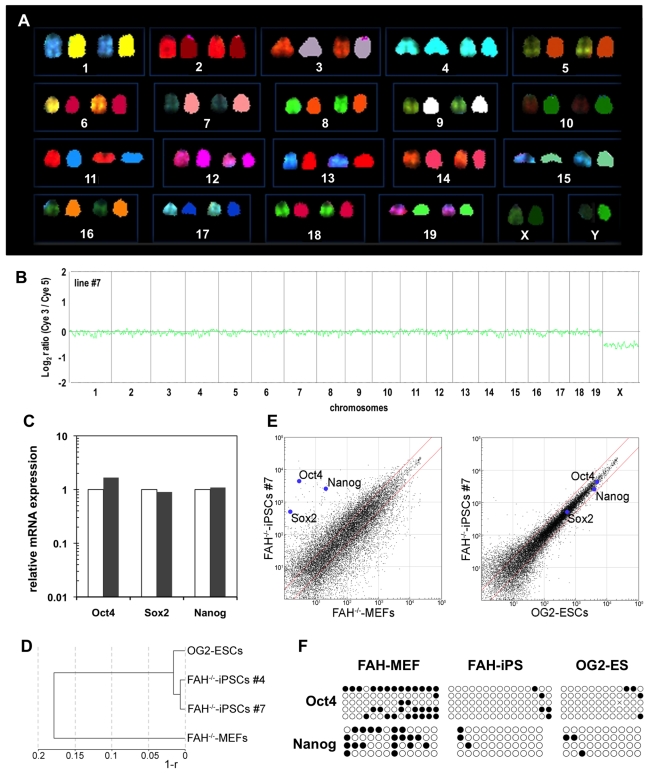
Molecular characterization of *FAH* ^−/−^-iPS cells. (A) SKY analysis of line #7 showed a normal male karyotype (40, XY) without any signs of chromosomal abnormalities. Each chromosome is depicted in original fluorescence color (left) and after classification (right). (B) Array CGH analyses of the male FAH^−/−^–iPS cell line #7 compared with the sex-mixed starting fibroblast (MEF) population further conformed its normal karyotype. (C) Quantitative RT-PCR for the pluripotency factors *Oct4*, *Sox2*, and *Nanog* in *FAH* ^−/−^-iPS cells compared with OG2 ES cells (normalized to 1). (D) Hierarchical clustering dendrogram of *FAH* ^−/−^-iPS cell lines based on global expression profile similarity (r: linear correlation coefficient). (E) Scatter plot of global gene expression data comparing *FAH* ^−/−^-iPS cells with *FAH* ^−/−^-MEFs (left panel) and *FAH* ^−/−^-iPS cells with ES cells (right panel). (F) Methylation status of *Oct4*- and *Nanog*-promoter regions in fibroblasts (starting cells: *FAH* ^−/−^-MEFs), *FAH* ^−/−^-iPS cells, and OG2 ES cells. Black circles depict methylated CpG islets; white circles show unmethylated CpG islets.

Fetal fibroblasts obtained from pregnant *FAH* ^−/−^ mice on 13.5 days post-coitum (dpc) were transduced with gamma-retroviral vectors expressing *Oct4*, *Sox2*, *Klf4*, and *c-Myc* to generate *FAH* ^−/−^-iPS cells. Five days after retroviral transduction, *FAH* ^−/−^ cells were replated on mouse embryonic fibroblast (MEF) feeder cells under mouse embryonic cell culture conditions. Within 9–14 d of transduction, we observed various distinct cell colonies. Based on morphological criteria, we chose 16 colonies, from which we established 8 iPS cell lines, of which we used two in our study (#4 and #7). These iPS cells stained uniformly positive for alkaline phosphatase activity (line #7 in Figure 1A,B) and expressed the pluripotency-associated markers Oct4, Sox2, and SSEA1 (Figure 1C–1K). Both iPS cell lines were male with a normal karyotype and did not show any detectable chromosomal translocations as shown by spectral karyotyping (SKY), which allows for detection and identification of structural chromosome aberrations with a resolution of approximately 2 Mb (Figure 2A) [44]. These results were confirmed using comparative genome hybridization (array CGH), demonstrating no detectable sub-chromosomal aberrations in line #7, compared with the starting fibroblasts (Figure 2B). As the latter contained mixed cells from both female and male fetuses, the male iPS cell line #7 depicts a relative loss of X-chromosome signals. It is clear that these are iPS cells, as we have never had any *FAH* ^−/−^ ES cells in our laboratory as a potential contamination source, and we have also ruled out contamination with *other* pluripotent stem cells in our lab by genotyping the *FAH* ^−/−^ allele, as depicted in Figure S1A. Furthermore, genomic integration of the retroviral vectors was confirmed, and individual gene copy numbers for the reprogramming vectors were determined by quantitative PCR using specific primer sets and by normalizing to 18S ribosome gene copy numbers (Table S1). We detected 12 and 8 retroviral insertions for iPS cell lines #4 and #7, respectively.

Next, we investigated the silencing of the 4 reprogramming factors in the established *FAH* ^−/−^-iPS cell line #7 (passage 9) compared with cells harvested 3 d after retroviral transduction with the 4 reprogramming factors (Figure S1B). Exogenous pMX retroviral–mediated expression of *Oct4*, *Klf4*, and *c-Myc* was markedly reduced to background levels, and retroviral *Sox2* expression was downregulated to less than 1.8% of its initial expression level 3 d after transduction.

However, quantitative real-time–PCR (qRT-PCR) expression analyses revealed that the iPS cells expressed endogenous *Oct4*, *Nanog*, and *Sox2* at levels similar to those of OG2 ES cells (Figure 2C). Global transcriptome analysis demonstrated the complete reprogramming of *FAH* ^−/−^ fibroblasts into induced pluripotent (*FAH* ^−/−^-iPS) cells, whose expression profile highly correlated with that of ES cells (r_lin_ = 0.98; Figure 2D,E). Thus, the subtle differences in gene expression levels were similar to those of independently derived ES cell lines. Finally, DNA methylation analysis of CpG-enriched islets in the *Oct4* and *Nanog* promoter regions (Figure 2F) demonstrated strong hypomethylation in both pluripotent cell types (*FAH* ^−/−^-iPS and OG2 ES cells), which indicates that these key factors play a role in the transcriptional network of cellular pluripotency.

### In Vivo Potential of *FAH* ^−/−^-iPS Cells

**Figure 3:**
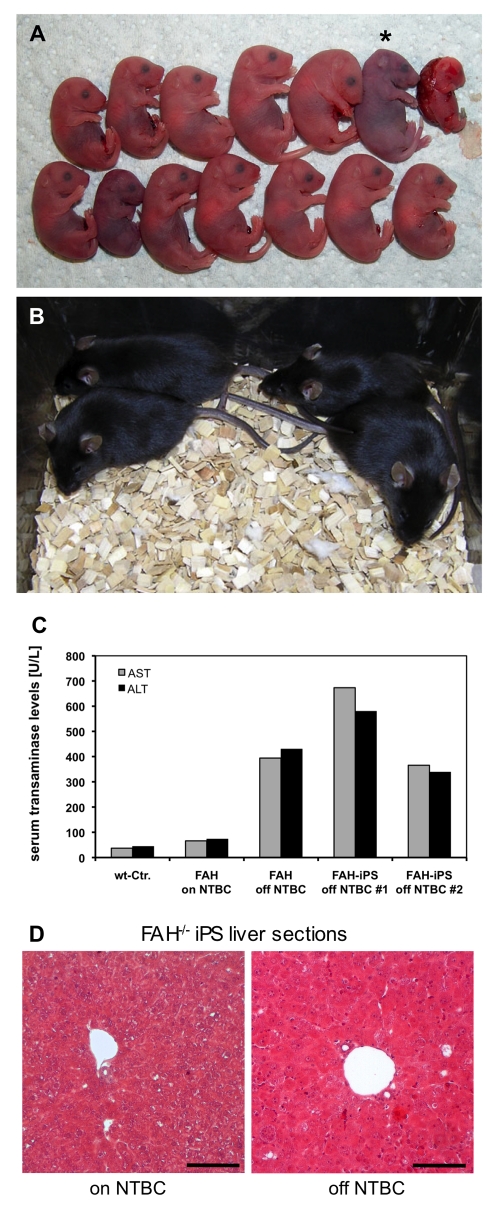
Viable mice derived from disease-specific *FAH* ^−/−^-iPS cells via tetraploid embryo complementation. (A) Neonatal *FAH* ^−/−^-iPS cell–derived pups delivered by caesarean section on E19.0. In this particular experiment, 13 pups showed normal full-term development, of which only one failed to initiate breathing (*). (B) *FAH* ^−/−^-iPS mice demonstrated normal postnatal development upon rescue of the underlying metabolic defect by NTBC supplementation. (C) Transaminase activity of AST (grey bars) and ALT (black bars) in the serum of untreated *FAH* ^−/−^ mice (off NTBC) indicated severe liver damage in *FAH* ^−/−^-iPS mice #1 and #2, as well as in *FAH* ^−/−^ control mice, compared with wild-type mice and *FAH* ^−/−^ mice treated with NTBC (normal levels for both). (D) H&E staining depicts only mild damage in treated *FAH* ^−/−^-iPS mouse liver (“on” NTBC) but severe damage in the liver of mice after NTBC withdrawal (“off” NTBC), as indicated by swollen and pale hepatocytes as well as by inflammatory infiltrations, which confirmed the unaffected diseased phenotype of *FAH* ^−/−^-iPS cell–derived mice, compared with the original *FAH* ^−/−^ mice. Scale bars, 100 µm.

The most robust proof for pluripotency of a given stem cell line is its ability to complement for the defective rudimentary inner cell mass of tetraploid mouse embryos (Figure S2A–S2F). In contrast to all previous studies, in which live pups were generated by injecting iPS cells into tetraploid blastocysts with an efficiency of 19%, 13%, 3.5%, and 1% [30]–[33], we applied the aggregation method using tetraploid 8-cell embryos and gene-corrected iPS cells. Using both *FAH* ^−/−^-iPS cell lines #4 and #72, we were able to generate fully developed pups on E19.0, when we performed caesarean section delivery. We obtained 39 (6.1%) fully developed live pups on E19.0 from 644 embryo aggregates with line #4 in nine individual experiments, but 21 of the pups failed to start breathing. Of the 18 pups that started breathing, only one survived for 3 wk and exhibited marked growth retardation. In contrast, iPS line #7 gave rise to many more viable mice in three individual sets of experiments. From a total number of 151 transferred aggregates, we obtained 43 fully developed male, non-chimeric (by coat color) pups on E19.0 at a surprisingly high efficiency of 28.5% (range, 21.2%–40.0%; see Figure 3A for a subset of one experiment). A total number of 34 pups started breathing after caesarean section delivery, of which 20 (13.2%) survived to adulthood (Table 1; Figure 3B). Most of the neonatal iPS cell–derived mice were shown, by PCR genotyping, to have entirely originated from the iPS cells. After 35 cycles of PCR, the wild-type *FAH* allele was detected in seven of 35 pups from line #4 and in 5 of 28 pups from line #7, and the faint intensity of the band indicated a very minor contribution of wild-type 4n cells in these mice (Figure S2G). Of the 11 adult iPS mice genotyped, 10 were found to be derived solely from iPS cells. Two of these 10 mice were mated with wild-type CD1 female mice to assess their fertility. We found that both male mice were fertile, and all the 38 F1 pups obtained contained the *FAH*-knockout allele (Figure S2H). In general, iPS cell–derived mice in the present study were supplemented with NTBC (2-(2-nitro-4-fluoromethylbenzoyl)-1,3-cyclohexanedione) in the drinking water [45] to prevent liver failure. To evaluate the diseased phenotype, a subset of four mice was challenged by NTBC withdrawal. As expected, these mice showed progressive loss of body weight, liver damage, and general lethargy within 14 d of NTBC withdrawal. Elevated serum levels of aspartate aminotransferase (AST, grey bars) and alanine aminotransferase (ALT, black bars) confirmed the disease-specific phenotype (Figure 3C), with AST and ALT levels comparable to those for original *FAH* ^−/−^ mice “off” NTBC. *FAH* ^−/−^ mice, which were “on” NTBC, did not show elevated transaminase levels compared with wild-type mice. Further histological analyses by hematoxylin and eosin (H&E) staining of the liver tissue of the iPS cell–derived mice showed severe liver damage, as indicated by swollen and pale hepatocytes as well as by inflammatory infiltrations in the livers of mice without NTBC treatment (Figure 3D), whereas only subtle pathologic changes were observed in the livers of the iPS cell–derived mice “on” NTBC. These findings clearly demonstrate that the iPS cell–derived mice faithfully replicated the phenotype of the founding *FAH* ^−/−^ mice.

**Table 1:** Experimental summary of tetraploid complementation of *FAH* ^−/−^-iPS cells compared with OG2 ES cells.

Cells Tested	Experiment	Passage Number	Aggregates Transferred		Full-Term Pups	on E19		Survived to Adulthood
				Breathing	Not Breathing	Dead	Total	
FAH–/–-iPS line #4	1	7	26	0	2	0	2	0
	2	8	88	2	3	0	5	0
	3	8	63	0	1	0	1	0
	4	10	128	2	1	0	3	0
	5	7	70	2	0	0	2	0
	6	8	83	5	1	1	7	0
	7	8	98	3	4	1	8	1
	8	5	38	1	4	0	5	0
	9	7	50	3	1	2	6	0
	**Total**		**644**	**18**	**17**	**4**	**39 (6.1%)**	**1 (0.16%)**
FAH–/–-iPS line #7	1	7	45	12	1	0	13	4
	2	7	40	13	3	0	16	9
	3	9	66	9	5	0	14	7
	**Total**		**151**	**34**	**9**	**0**	**43 (28.5%)**	**20 (13.2%)**
OG2 ES	1	7	36	10	1	1	12	8
	2	7	50	10	4	0	14	7
	**Total**		**86**	**20**	**5**	**1**	**26 (30.2%)**	**15 (17.4%)**

### Genetic Correction of *FAH* ^−/−^-iPS Cells and Generation of Viable Mice from Gene-Corrected iPS Cells

**Figure 4:**
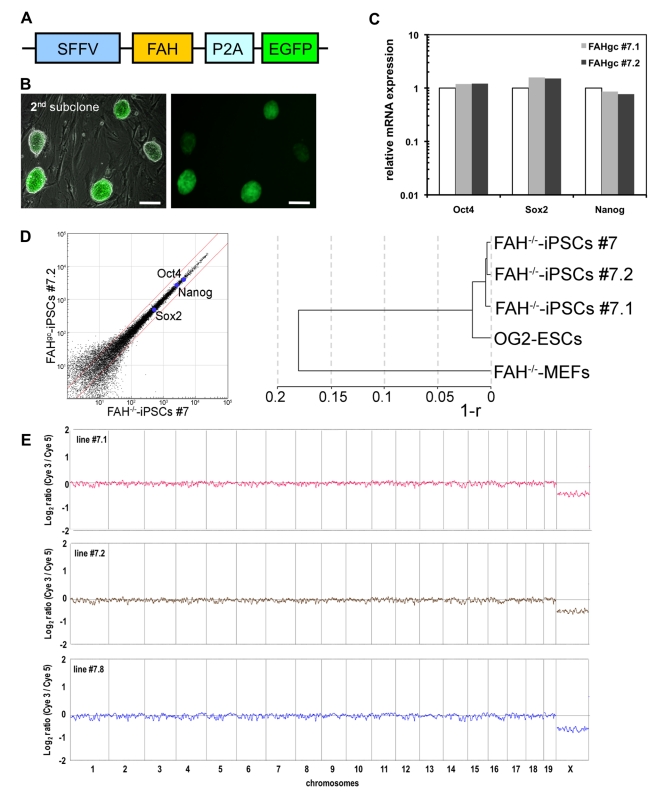
Genetic correction of *FAH* ^−/−^-iPS cells. (A) Design of the lentiviral construct for gene correction of *FAH* ^−/−^ cells: An intact *FAH* cDNA was expressed in conjunction with EGFP, linked by a 2A-peptidase motif (P2A) for coexpression, under the transcriptional control of the spleen focus-forming virus (*SFFV*) promoter. (B) After transduction of *FAH* ^−/−^-iPS cells in single-cell suspension, colonies showing homogenous EGFP expression were subcloned. A second subcloning step was applied to ensure clonality of the gene-corrected *FAH* ^gc^-iPS cell lines, as depicted for line #7.2. Scale bars, 100 µm. (C) Quantitative RT-PCR for the pluripotency factors *Oct4*, *Sox2*, and *Nanog* in *FAH* ^gc^-iPS cells (#7.1 and #7.2) compared with OG2 ES cells (normalized to 1). (D) Scatter plot of global gene expression data comparing *FAH* ^gc^-iPS cell line #7.2 with *FAH* ^−/−^-iPS cell line #7, and dendrogram of the indicated samples based on global expression profile similarity (r: linear correlation coefficient). (E) Array CGH analyses of the male gene-corrected FAH^gc^–iPS cell lines #7.1, #7.2, and #7.8 showed no detectable chromosomal aberrations, compared with the sex-mixed starting fibroblast (MEF) population.

For the genetic correction of the *FAH* mutation in *FAH* ^−/−^-iPS cells, we first constructed a lentiviral vector coexpressing an intact wild-type *FAH* cDNA and the reporter gene *EGFP* sequences linked by a 2A-peptide motif under the transcriptional control of a strong ubiquitously expressed viral promoter, the spleen focus-forming virus (*SFFV*) promoter (Figure 4A). After transducing *FAH* ^−/−^-iPS cell line #7 of an early passage number (p7), we performed two rounds of subcloning to derive pure gene-corrected *FAH* (*FAH* ^gc^)-iPS cell lines by picking colonies with homologous EGFP expression and typical ES cell morphology (Figure 4B). Quantitative RT-PCR analyses for *Oct4*, *Nanog*, and *Sox2* expression (Figure 4C) confirmed the pluripotent phenotype of the two *FAH* ^gc^-iPS cell lines tested (#7.1 and #7.2). As depicted in Figure 4D, global expression profiles were unaffected by the gene correction procedure and all three tested iPS cell lines clustered very closely—*FAH* ^gc^-iPS cell line #7.2 was slightly closer to the parental iPS cell line #7 than was line #7.1. Both gene-corrected cell lines (#7.1 and #7.2) and another derivative (#7.8) did not exhibit detectable subchromosomal aberrations and did not show aberrant copy number variations, compared with the starting fibroblasts, as determined by array CGH analyses (Figure 4E).

**Figure 5:**
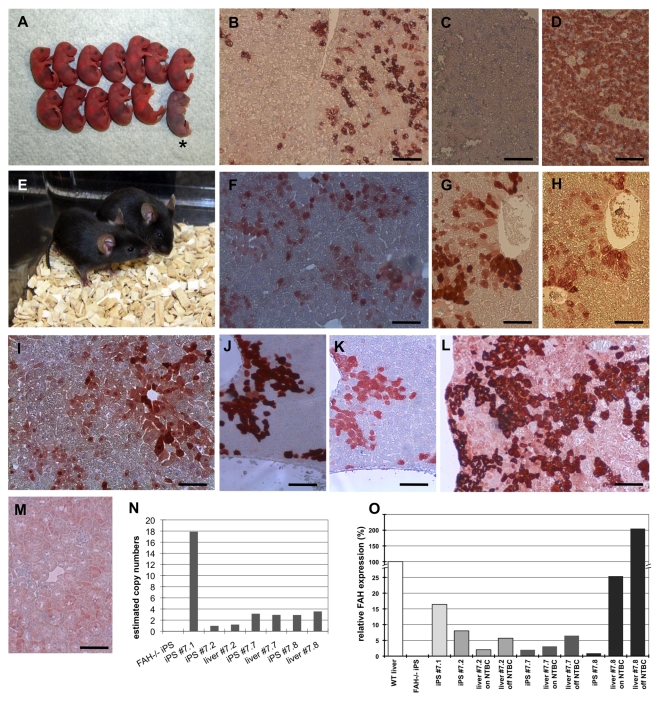
Viable mice derived from gene-corrected *FAH* ^gc^-iPS cells via tetraploid embryo complementation. (A) A representative litter of *FAH* ^gc^-IPS cell–derived neonatal pups. One of the pups failed to initiative breathing (*) after caesarean section delivery on E19.0. (B) Immunohistological staining for FAH demonstrated that about 20% of the hepatocytes in a newborn's liver expressed the FAH protein. (C) *FAH* ^−/−^ liver tissue without gene correction was used as a negative control, confirming the specificity of the antibody staining. (D) Wild-type liver was used as a positive control to confirm the sensitivity of the FAH immunohistochemistry. All hepatocytes, but no endothelial or blood cells, stained positive for FAH. (E) Two healthy #7.2 (*SFFV* promoter) *FAH* ^gc^-iPS cell–derived mice under supplementation with NTBC. (F and G) FAH staining on the liver of these mice in (E) indicated FAH expression in about 25% of the liver cells. (H) Immunohistochemistry counterstaining for EGFP demonstrated that all FAH-positive cells in panel (G) also stained positive for EGFP. (I) FAH staining in #7.2 (*SFFV* promoter) *FAH* ^gc^-iPS cell–derived mouse liver 5 wk after NTBC withdrawal showed FAH expression in most of the liver cells. (J) FAH staining in #7.8 (*TTR* promoter) *FAH* ^gc^-iPS cell–derived mouse liver indicates FAH expression in about 15% of the liver cells. (K) Immunohistochemistry counterstaining for EGFP demonstrates that all FAH-positive cells in panel J also stained positive for EGFP. (L) FAH staining in #7.8 (*TTR* promoter) *FAH* ^gc^-iPS cell–derived mouse liver 4 wk after NTBC withdrawal indicates FAH expression in most of the liver cells. (M) Absence of FAH-positive staining in a kidney sample of iPS line #7.8–derived mice confirmed the tissue specificity of the hepatic *TTR* promoter. (N) Estimated gene copy numbers of the *FAH-P2a-EGFP* construct in both *FAH* ^gc^-iPS cell lines #7.1 and #7.2 as well as in liver tissue of an *FAH* ^gc^-iPS cell #7.2–derived mouse. (O) *FAH* mRNA expression levels relative to adult liver tissue (set as 100%) in *FAH* ^gc^-iPS cell lines and in liver samples of an *FAH* ^gc^-iPS cell–derived mouse (“on” and “off” NTBC). Scale bars, 100 µm.

Next, we used tetraploid embryo complementation to evaluate the developmental potential of the gene-corrected iPS cells (Table 2). We transferred 172 aggregates comprising tetraploid embryos and *FAH* ^gc^-iPS cells into pseudopregnant mothers and obtained 41 fully developed male pups (23.8%) on E19.0 by caesarean section delivery. Most of the pups generated with *FAH* ^gc^-iPS cell line #7.1 failed to start breathing and showed severe fetal abnormalities, with only one mouse surviving to adulthood. However, 17 breathing *FAH* ^gc^-iPS mice were obtained with *FAH* ^gc^-iPS cell line #7.2 (Figure 5A). Immunohistochemical analyses of liver sections from the nonbreathing neonatal pups of line #7.2 demonstrated the presence of the FAH protein in a minority of the liver cells (Figure 5B), indicating silencing of the *FAH* transgene in most of the *FAH* ^gc^-iPS–derived cells. The absence of nonspecific staining in the *FAH* ^−/−^ livers and the presence of evenly distributed staining in wild-type livers confirmed the specificity and sensitivity of the antibody, respectively (Figure 5C,D).

**Table 2:** Experimental summary of *FAH* ^gc^-iPS cell tetraploid complementation.

Cells Tested	Experiment	Passage Number	Aggregates transferred		Full-Term Pups	on E19		Survived to Adulthood
				Breathing	Not Breathing	Dead	Total	
Line #7.1 (SFFV)	1	11	50	1	5	1	7	0
	2	12	27	4	9	0	13	1
	**Total**		**77**	**5**	**14**	**1**	**20 (26.0%)**	**1 (1.3%)**
Line #7.2 (SFFV)	1	11	39	5	3	0	8	2
	2	12	56	12	1	0	13	4
	**Total**		**95**	**17**	**4**	**0**	**21 (22.1%)**	**6 (6.3%)**
**#7.7 (TTR)**	**1 (Total)**	**13**	**30**	**15**	**2**	**0**	**17 (56.7%)**	**6 (20.0%)**
**Line #7.8 (TTR)**	**1 (Total)**	**13**	**30**	**19**	**2**	**0**	**21 (63.3%)**	**11 (36.7%)**

Based on these results, we decided to maintain the foster mothers with NTBC supplementation to ensure the subsequent development of the *FAH* ^gc^-iPS cell–derived neonatal mice. Of the 17 breathing mice, 13 were initially accepted and fed by the foster mothers, but of these, seven died within the first week of birth. Nevertheless, six mice survived to adulthood (Figure 5E) and were healthy for more than 12 mo, without any evidence for tumor formation. Two of these six mice were kept without NTBC supplementation during month 4 to month 12, suggesting that the diseased phenotype was effectively rescued. In a liver biopsy (25% hepatectomy) from a 7-wk-old *FAH* ^gc^-iPS cell–derived mouse, we observed multiple cells that stained positive for FAH by immunohistochemistry (Figure 5F). However, in a significant number of cells no FAH protein could be detected. To confirm that the FAH-positive cells were not derived from derivatives of the host embryo or other wild-type cells, we assessed consecutive liver sections for FAH (Figure 5G) and EGFP (Figure 5H) by immunohistochemistry. We found that all cells that stained positive for FAH also expressed EGFP, indicating that they were derived from the gene-corrected iPS cells.

### Functional Restoration of the Diseased Phenotype

To assess the efficacy of gene correction, we challenged the gene-corrected iPS cell–derived mice by NTBC withdrawal. If the diseased phenotype were fully restored, we would expect that the non-FAH–expressing hepatocytes would all gradually die and that the healthy FAH-expressing hepatocytes would proliferate and repopulate the liver. Five weeks after NTBC withdrawal, all mice appeared to be healthy, with no signs of *FAH* ^−/−^-associated symptoms—that is, they were NTBC independent. Additionally, by surgical liver biopsy, we confirmed the expansion of FAH-expressing hepatocytes in the livers of these mice. Furthermore, 50%–70% of the hepatocytes in these biopsy sections stained positive for FAH (Figure 5I), compared with 25% FAH-positive hepatocytes in *FAH* ^gc^–iPS cell-derived mice with NTBC supplementation (Figure 5F). This finding reflects a 2–3-fold increase in FAH-positive hepatocytes in mice after NTBC withdrawal.

### Gene Correction of *FAH* ^−/−^-iPS Cells with Mammalian Tissue-Specific Promoter

We assumed that mammalian tissue-specific promoter sequences might be less susceptible to silencing, but more susceptible to sustained transgene expression than viral promoter motifs. Therefore, we generated two new gene-corrected iPS lines (*FAH* ^gc^–iPS cells #7.7 and #7.8) by exchanging the viral *SFFV* promoter cassette with a murine transthyretin (*TTR*) promoter sequence in the lentiviral gene therapy vector. As both the *EGFP* and *FAH* transgenes were driven by this liver-specific promoter, the successfully transduced iPS cells could not express EGFP at the pluripotent stage and thus could not be subcloned based on EGFP expression. Alternatively, we randomly picked iPS cell colonies, expanded them individually, and then screened for *TTR-FAH-2A-GFP* transgene integration by PCR. As a result, we could only use the *FAH* ^gc^-iPS cell lines (#7.7 and #7.8) of passage 13 or more for subsequent experiments. By applying array CGH analyses, we confirmed the absence of relevant chromosomal abnormalities in line #7.8, as depicted in Figure 4E. Surprisingly, both lines were robust in generating all iPS cell–derived mice. We obtained full-term pups at an efficiency of 56.7% and 63.3% of transferred aggregates generated with #7.7 and #7.8 iPS cells, respectively (Table 2). Mice derived from #7.8 iPS cells showed robust expression of the *FAH-2A-EGFP* transgene in the liver (Figure 5J,K) in a small portion of hepatocytes as analyzed by FAH and EGFP immunohistochemistry, respectively, but again the *TTR* promoter–driven transgenes were silenced in most of the hepatocytes. This was even more evident in mice derived from #7.7 iPS cells, with very few FAH-positive cells detected. In a subsequent experiment, some newly weaned mice were kept without NTBC supplementation and analyzed 4 wk later. All mice derived from *FAH* ^gc^-iPS #7.7 appeared very weak after NTBC withdrawal and presented characteristics of liver failure with very few FAH-positive liver cells. In contrast, 50%–70% of cells stained positive for FAH in the livers of mice derived from #7.8 iPS cells (Figure 5L), similar to observations with the *SFFV* promoter-driven construct. Up to now, 11 mo after NTBC withdrawal, two mice derived from #7.8 iPS cells are still in good health, with no signs of any *FAH* ^−/−^-associated symptoms. These findings suggest that the number of FAH-expressing cells increased after NTBC withdrawal—that is, the regenerative stimulus—and the cluster-like appearance of the FAH-expressing cells (Figure 5L) indicated the proliferation of functionally corrected cells, rather than the reactivation of the silenced transgenes in FAH-negative cells, which would have resulted in a more stochastic distribution of single FAH-expressing cells. To confirm the tissue-specific expression of the *TTR* promoter–driven transgene, we demonstrated the absence of FAH expression in other tissues, such as spleen and kidney, by immunohistology (Figure 5M).

Furthermore, the copy number of the *FAH* transgene in all four *FAH* ^gc^-iPS cell lines and their liver tissue derivatives was determined by quantitative PCR with the *FAH-2A-GFP* genomic DNA construct (Figure 5N). *FAH* ^gc^-iPS cell line #7.1 had 18 integrations of the *FAH-2A-GFP* transgene, line #7.2 had only one insertion, and lines #7.7 and #7.8 each had three insertions of the gene-correction vector. The genomic DNA obtained from the *FAH* ^gc^-iPS cell–derived liver tissue contained the same transgene number, which also ruled out any major contamination with tetraploid host embryo cells or non-corrected *FAH* ^−/−^-iPS cells in the livers. *FAH* mRNA expression (Figure 5O) in the gene-corrected liver tissues and in the parental *FAH* ^gc^-iPS cells was also analyzed. With respect to the first set of experiments using the *SFFV* promoter–driven construct, a constitutive *FAH* expression was observed at levels of 8% (wild-type liver tissue was set as 100%) in line #7.2. Consistent with the *FAH* silencing in about 75% of the differentiated liver cells (Figure 5F), the overall *FAH* mRNA expression in the liver of an *FAH* ^gc^-iPS cell–derived mouse supplemented with NTBC dropped to 2.0%—that is, to 25% of the expression level in line #7.2. The challenge of NTBC withdrawal in FAH-positive cells resulted in a 2.8-fold increase in *FAH* gene expression (5.7%) in *FAH* ^gc^-iPS cell line #7.2–derived liver 5 wk after NTBC withdrawal. As expected, *FAH* expression in the *FAH* ^gc^-iPS cell lines #7.7 and #7.8, with the liver-specific *TTR* promoter, was virtually undetectable. Consistent with our immunohistological observation, the *FAH* transgene was only expressed at very low levels in the whole livers of line #7.7–derived mice. But in liver samples from line #7.8–derived mice, *FAH* was expressed at a level of 25% relative to wild-type liver in unchallenged NTBC-treated mouse livers. In the NTBC withdrawal group, the relative *FAH* expression was 207%, which further provides strong evidence that the FAH-positive cells were functionally responsive to the regenerative stimulus, NTBC withdrawal, and increased in numbers by roughly 4-fold. Two of these mice survived for more than 12 mo, with no FAH^−/−^ phenotype, demonstrating the full functional restoration of the *FAH* deficiency in the gene-corrected iPS cell line #7.8.

## Discussion

Although entirely mouse iPS cell–derived mice have been generated [30]–[33], iPS cells carrying a disease-specific genetic defect have not yet been shown to retain full pluripotency upon genetic correction and prolonged propagation, as well as to “pass” stringent tests for pluripotency. In our study, we were able to generate metabolic liver disease–specific iPS cells from fetal fibroblasts of *FAH* ^−/−^ mice [34] and to maintain various clonal lines that show all phenotypic hallmarks of pluripotent stem cells, as seen for line #7 in Figures 1 and 2.

For the initial tetraploid embryo complementation experiments, we chose two independent *FAH* ^−/−^-iPS cell lines (#4 and #7), which gave rise to different results as summarized in Table 1. The *FAH* ^−/−^-iPS cell line #7 yielded up to a 28.5% efficiency in generating full-term pups by aggregation of iPS cells with a tetraploid morula in a sandwich manner, which was even hardly achievable with advanced protocols using normal ES cells [46]. The gene-corrected iPS cells of line #7.2 gave rise to 21 (22.1%) full-term pups, of which 6 (6.3%) developed into adult mice. Using the gene-corrected iPS lines #7.7 and #7.8, the efficiency of obtaining full-term pups (and adult mice) was even higher, reaching 56.7% (and 20.0%) and 63.3% (and 36.7%), respectively, probably due to the multiple selection steps during the gene transfection process. This efficiency is in the very same high range of normal ES cell aggregates and it is much higher than the efficiency for iPS cells per recent reports: 0.53% [31], 3.5% [32], and 2% and 7% [30]. A possible explanation for the higher efficiency is that, unlike all these reports, we did not use the blastocyst injection method for generating iPS cell–derived mice, but exclusively used the morula aggregation method with selected colonies of iPS cells that showed the most intact colony morphology. We speculate that other groups have used different criteria to identify and process iPS or ES cells. As the presence of discriminating markers [33] for bona fide pluripotent iPS cells have been suggested to correlate with pluripotency, it is unclear whether remnants of an epigenetic memory account for the markedly less efficient full-term pup development in the other tetraploid embryo complementation studies using fibroblast-derived iPS cells [30]–[32]. Nevertheless, our results demonstrate that morula aggregation is a simple and highly efficient approach to generate entirely iPS cell–derived mice via iPS cell/tetraploid embryo complementation.

With our data, we clearly demonstrated that our murine iPS cells (line #7 and its derivatives) were equivalent to ES cells, by the most stringent test for pluripotency—tetraploid embryo complementation. The reduction in the number of reprogramming factors for the generation of iPS cells [47]–[49] is considered to be beneficial for iPS cell generation and is expected to obviate limitations related to chromosomal transgene integrations. However, despite numerous attempts using our 2-factor and 1-factor iPS cells [50], we have not yet been able to successfully complete tetraploid embryo aggregation. It would be interesting to discover the underlying molecular differences between the lines, which would lead to a better understanding of pluripotency and might allow for the identification, by simple marker gene expression, of high-quality iPS cell lines for future clinical applications. In our set of experiments, the gene expression profile of the less successful *FAH* ^−/−^-iPS cell line #4 clustered very closely to that of the high-quality line #7 (Figure 2D), and we only detected significant differences in very few genes (*Jarid1d*, *Erdr1*, *Eif2s3y*). However, a comparison of only these two independent iPS cell lines (#4 and #7) may not identify differentially expressed genes and predict fully pluripotent iPS cells that can pass the tetraploid embryo complementation test. Aiming to identify such discriminating transcripts, the *Dlk1-Dio* cluster (on the long arm of chromosome 12) was recently reported to carry a few imprinted genes that exhibit a distinct differential expression pattern in fully pluripotent ES cells compared with iPS cells [33], with these genes suggested as candidate markers for selection of high-quality iPS cell lines. However, in our study, we could not find significant differences in *Dlk1*, *Gtl2*, and *Rian* expression among our *FAH* ^−/−^-iPS cell lines, which actually all gave rise to full-term iPS cell–derived pups but supported various degrees of postnatal development. Postnatal developmental failure was observed in *FAH* ^−/−^-iPS line #4 and #7.1–derived mice. As an alternative explanation, the four additional insertions of the reprogramming factors found in line #4 (Table S1) and the high transgene copy number detected in line #7.1 may account for the different outcomes in our study.

The development of iPS cell–based transplantation approaches for the treatment for genetic disorders must comprise a gene-correction strategy for rescuing the diseased phenotype [51]. The genetic engineering of ES and iPS cells appears to be rather inefficient, but recently published studies on a targeted genetic engineering approach show promise [37],[52]. As outlined above, the safety issues associated with using electroporation to deliver large flanking regions for homologous recombination of an entire gene locus have not yet been addressed, as severe DNA double-strand breaks are expected [35],[36]. The more sophisticated tailored zinc finger nuclease–mediated homologous recombination is considered to be a versatile tool for gene therapy of genetic diseases with a single common mutation among patients, such as cystic fibrosis (Δ508, Phe508del), sickle cell anemia (Glu6Val), or α1-antitrypsin deficiency (Glu342Lys). However, various missense, nonsense, and splice-site mutations, along with small deletions in the FAH locus, have been reported among patients with tyrosinemia type I syndrome—a condition considered as representative for many orphan diseases. Unfortunately, the development of gene therapeutic strategies for these diseases has been relegated to academic research groups. For such disease entities, an additive gene transfer strategy provided by a stable integration of an intact gene copy is the preferred approach. Third-generation self-inactivating lentiviral vectors, which are significantly safer than conventional gamma-retroviral vectors, are now available for gene therapy applications [40],[41]. For example, lentiviral gene correction was successfully accomplished in fibroblast cells prior to generating Fanconi's anemia–specific iPS cells [17]. In the present study, we chose such a lentiviral vector with insertion of the intact *FAH* cDNA and EGFP sequences linked by a 2A-peptidase motif, which allowed for fluorescence–based selection of FAH-expressing iPS cells to generate pure clonal gene-corrected *FAH* ^gc^-iPS cell lines. To investigate the effects of the gene dosage on pluripotency, we analyzed two independent clonal lines—one with high EGFP expression and 18 copies of the *FAH* transgene (line #7.1) and one with moderate EGFP expression and only one copy of the transgene (line #7.2). When we performed tetraploid embryo complementation with cells of line #7.1, even though the efficiency of obtaining full-term neonatal iPS cell–derived pups was high (26.0%), most of the iPS cell–derived pups exhibited malformations and could not start breathing, and only one survived to adulthood (1.3%, Table 2). However, using line #7.2, we were able to obtain 17 breathing pups of 21 fully developed pups from 95 transferred aggregates (Figure 5A), of which 6 survived to adulthood. However, in contrast to this dramatic difference in postnatal developmental potential, we could not observe significant differences in the global gene expression pattern between lines #7.1 and #7.2, as depicted in the dendrogram in Figure 4D. The correlation between postnatal developmental failure and increased copy number of transgene integration indicated the presence of lentiviral genotoxicity, which deserves further study to identify differentially expressed genes or discriminating (epi)genetic marks among gene-corrected iPS cells and their relation to postnatal development.

The *FAH* ^−/−^ mouse model has been well established for analyses of gene therapy strategies [53]–[56] and cell transplantation approaches [57]–[59] of metabolic liver disorders, but to date, no ES cell–based approach has demonstrated success in rescuing the diseased phenotype. A very recent study took advantage of generating diploid chimeras of wild-type iPS cells and *FAH* ^−*/*−^ embryos to prove the ability of iPS cells to give rise to functional and transplantable hepatocytes in vivo [43]. Such an approach could not be used to establish a disease model from somatic cell–derived iPS cells or to investigate genetic manipulation for genetic repair in disease-modeled iPS cells. Using the lentiviral vector approach to express the intact *FAH* cDNA sequence in *FAH* ^−/−^ iPS cells, we observed silencing of the *FAH*-repair cassette in a significant proportion of the hepatocytes from fetal and adult iPS cell–derived mice (Figure 5B,F–L). Such silencing of the lentiviral gene repair construct was also reported for Fanconi's anemia–specific iPS cells [17]. However, when we challenged *FAH* ^gc^-iPS cell–derived mice by NTBC withdrawal, we found a clear increase in the number of FAH-positive hepatocytes (Figure 5I,L), and accordingly a 2.7-fold increase in the *FAH* gene expression level by quantitative RT-PCR (Figure 5O). The long-term survival, more than 12 mo, of these *FAH* ^gc^-iPS cell–derived mice without NTBC supplementation provided unequivocal evidence for the efficacy of the gene correction approach in our study. The coexpression of EGFP in all FAH-expressing cells (Figure 5G&H, J&K) excluded the possibility that *FAH* ^−*/*−^ phenotypes in the *FAH* ^gc^-iPS cell–derived mice were actually rescued by the residual wild-type cells from the host 4n embryos. With these findings, we demonstrate for the first time that gene-corrected iPS cells could give rise to hepatocytes that both respond to proliferative stimuli and are capable of restoring liver function. However, our findings also suggest that lentiviral gene repair constructs introduced into iPS cells are highly susceptible to silencing. This undesirable silencing was evident with either a strong viral promoter motif (*SFFV*) or a mammalian tissue-specific promoter sequence (*TTR*), and it could be overcome if a selective pressure was present for the transgene-expressing cells.

In conclusion, metabolic liver disease–specific iPS cells obtained from reprogrammed somatic cells can be gene corrected to generate entirely iPS cell–derived healthy mice using the highly efficient tetraploid embryo complementation approach. With our data, we demonstrate that a life-threatening genetic disease can be treated via an iPS cell approach. As this approach can lead to a functional organism, we would expect that fully functional cells could be generated and used for transplantation purposes.

## Materials and Methods

### Ethics Statement

All animal experiments were performed according to protocols approved by the local authorities. All our mice had free access to food and water and were handled in accordance with institutional guidelines.

### Cell Culture and Generation of *FAH*-iPS Cells

Mouse embryonic stem cells (OG2 ES) carrying an Oct4-GFP reporter construct [60] and induced pluripotent stem (iPS) cells were cultured according to standard conditions [61]. pMX-based retroviral vectors encoding the mouse complementary DNAs for *Oct4*, *Sox2*, *Klf4*, and *c-Myc* [2] were cotransfected with packaging-defective helper plasmids into 293T cells using FuGENE 6 Transfection Reagent (Roche, Mannheim, Germany) as described previously [47]. Virus-containing supernatants were collected 48 h later. Fetal fibroblasts obtained from pregnant C57BL/6 *FAH* ^−/−^ mice (provided by Arndt Vogel, Hannover Medical School) on E13.5 were seeded at a density of 5×10^4^ cells per well of a 6-well plate and incubated with virus-containing supernatants of the 4 factors (1∶1∶1∶1) supplemented with 6 µg ml^−1^ protamine sulfate (Sigma, Munich, Germany) for 24 h.

### Characterization of iPS Cells

*FAH* ^−/−^-iPS cells grown on feeder cells were fixed in 80% (v/v) methanol (Sigma) for 30 min, before staining (overnight at 4°C) with mouse anti-Oct4, rabbit anti-Sox2 (1∶200; Santa Cruz Biotechnology, Santa Cruz, CA), or biotinylated mouse anti-SSEA1 (1∶300; eBioscience, San Diego, CA). Nuclei were labeled with DAPI (Sigma) and Cy3-conjugated rabbit anti-mouse IgG; FITC-conjugated goat anti-rabbit IgG (1∶300; Jackson ImmunoResearch, Newmarket, Suffolk, UK) and FITC-Streptavidin (1∶200; eBioscience) were used as secondary reagents. Alkaline phosphatase staining was performed with Alkaline Phosphatase Detection Kit (Millipore, Billerica, MA) according to the manufacturer's instructions.

For spectral karyotyping (SKY), metaphase chromosomes were prepared using standard protocols. In brief, cells were treated with colcemid at a final concentration of 0.035 µg ml^−1^ overnight, incubated in 0.075M KCl for 20 min at 37°C, and fixed in a freshly prepared mixture of methanol:acetic acid (3∶1) at room temperature. The cell suspension was dropped onto glass slides in a climate chamber (Polymer, Kassel, Germany) at 22°C and 48% humidity. SKY analysis was performed as described previously [44]. Spectral images were acquired using an epifluorescence microscope equipped with an interferometer (SpectraCube ASI), a custom-designed optical filter, and the SkyView software (ASI).

Array CGH was performed using the Agilent oligonucleotide-based Mouse Genome Microarray Kit 4×180 k (G4839A, Agilent Technologies, Santa Clara, CA, USA). Labeling and hybridization of genomic DNA was performed according to the protocol provided by Agilent. Briefly; 1.5 µg of test DNA (FAH-iPS) and reference DNA (mixed FAH-MEF cells from male and female fetuses) were labeled by random priming using the Agilent Genomic DNA Labeling Kit Plus, test DNA with Cy3-dUTP, and reference DNA with Cy5-dUTP. Labeled products were purified by Amicon ultra-0.5 ml 30K filters (Millipore, Billerica, MA, USA), combined, and then mixed with Mouse Cot-1 DNA (50 µg), Agilent 10X Blocking Agent, and Agilent 2X Hybridization Buffer. This solution was hybridized to Agilent's 4×180 k Mouse Genome CGH microarray at 65°C with 20 rpm rotation for 40 h. Washing steps were performed according to the Agilent protocol. Microarray slides were scanned immediately using an Agilent microarray scanner. For image analysis, default CGH settings of Feature Extraction (FE) Software 10.10.1.1 (Agilent Technologies, Waldbronn, Germany) were applied. Output files from FE were subsequently imported into Agilent's CGH data analysis software, Genomic workbench 6.5. for DNA copy number analysis using ADM-2 (aberration detection module) algorithm set to threshold of 6.0 and aberration filter set to 10 probes with mean log2 ratio of 0.4.

*FAH* transgene copy numbers were determined using primers for the lentiviral backbone (forward: 5′-GAG GAG TTG TGG CCC GTT GT-3′; reverse: 5′-TGA CAG GTG GTG GCA ATG CC-3′). The retroviral-specific primers consist of a common sense primer (5′- CTT GAA CCT CCT CGT TCG AC-3′) located upstream of each transgene within the retroviral backbone and a gene-specific antisense primer (Oct4: 5′-CTC CGC AGA ACT CGT ATG C-3′; Sox2: 5′-GGG CTG TTC TTC TGG TTG C-3′; Klf4: 5′-ACG CAG TGT CTT CTC CCT TC-3′; and c-Myc: 5′-TCC TCG TCG CAG ATG AAA TAG-3′). For normalization, 18S ribosome gene copy number (200 copies in murine cells [62]) was used with the primer pair 5′-AGG GCA GGG ACT TAA TCA ACG C-3′ and 5′-GTT GGT GGA CCG ATT TGT CTG G-3′.

### Gene Expression Analyses

Total RNA was isolated using TRIzol reagent (Invitrogen, Karlsruhe, Germany) and the resultant RNA was subjected to DNase treatment and cDNA transcription (SuperScript III cDNA synthesis kit, Invitrogen) to perform qRT-PCR (StepOne plus Cycler, Applied Biosystems, Darmstadt, Germany). We used TaqMan Assays-on-Demand for *Oct4* (Mm00658129_gH), *Sox2* (Mm00488369_s1), *Nanog* (Mm02019550_s1), *Fah* (Mm01312827_m1), and *β-actin* (Mm00607939_s1) for normalization.

Total RNA samples to be hybridized on Illumina mouse-8 V2 expression BeadChips were processed using a linear amplification kit (Illumina TotalPrep RNA Amplification Kit, Ambion) generating biotin-labeled cRNA. After assessing its quality on a 2100 Bioanalyzer (Agilent), RNA was hybridized as recommended and using materials/reagents provided by the manufacturer. Background subtraction, data normalization (cubic spline algorithm), and global expression profile comparisons were carried out using BeadStudio. All raw and processed microarray data have been submitted to Gene Expression Omnibus (GEO) database (http://www.ncbi.nlm.nih.gov/gds) under the accession number GSE21343, in a MIAME-compliant format.

For real-time analysis of the pMX-driven expression of the four factors, RNA from iPS cells was extracted using the RNeasy Mini Kit (QIAGEN, Hilden, Germany), following the manufacturer's instructions. Complementary DNA synthesis was performed with the High Capacity cDNA Archive Kit (Applied Biosystems) following the manufacturer's instructions. Transcript levels were determined using the ABI PRISM 7900HT Sequence Detection System (Applied Biosystems) and custom oligonucleotides for the 5′-nuclease assay, which can amplify the viral transcripts but not the endogenous ones [47]. The expression of the viral factors was normalized to the endogenous *Hprt1* gene (Mm00446968_m1, from Applied Biosystems), using the ΔΔC_T_ method.

DNA methylation was analyzed by bisulfite treatment and sequencing of the promoter regions of *Oct4* and *Nanog* as described previously [63]. For gene copy number analyses, genomic DNA was isolated using GenElute Mammalian Genomic DNA Miniprep Kit (Sigma) according to the manufacturer's instructions. Quantitative PCR was performed on three technical replicates using PCR Master-Mix (Peqlab Biotechnology) supplemented with SYBR green (Lonza, Rockland, ME) on a StepOne plus Cycler (Applied Biosystems).

### Analyses of the *FAH* ^−/−^ Phenotype

*FAH* ^−/−^ mice were maintained by supplementation of drinking water with 2-(2-nitro-4- trifluoromethylbenzoyl)-1,3-cyclohexanedione (NTBC, 4 mg ml^−1^, from Swedish Orphan International, Stockholm, Sweden). For determination of the liver enzyme activity, heparinized blood samples were collected from mice and centrifuged at 3,000 rpm for 10 min. Sera were stored in a −20°C refrigerator. ALT and AST levels were analyzed in a routine laboratory with an automatic analyzer (Vitros 5.1, Ortho, France).

For liver biopsy, mice were anesthetized with isoflurane before a partial hepatectomy was performed in which 25% of liver was removed. The left lateral lobe was tied to block the blood supply before surgical removal of the intact lobe.

For immunohistochemistry analyses, paraffin-embedded liver tissue was processed for H&E staining according to standard protocols. For FAH staining, endogenous peroxidase was blocked with 3% hydrogen peroxide in methanol for 10 min, followed by antigen retrieval in target retrieval solution (Dako, Glostrup, Denmark) for 20 min in a water bath at 98°C. Next, the sections were serially incubated with avidin/biotin block and 20% goat serum (Vector Laboratories, Burlingame, CA) for 15 min at room temperature, and then with primary antibody rabbit polyclonal anti-FAH (IgNex, 1∶400) or primary antibody rabbit polyclonal anti-GFP (Invitrogen, 1∶250) overnight at 4°C. The sections were then serially incubated with secondary antibody biotinylated goat anti-rabbit (1∶700) as well as EliteA and B (Vector Laboratories) for 1 h at room temperature, to allow enzyme complex formation and thus color development with 3-amino-9-ethylcarbazole (Dako). Sections were counterstained with Gill's No. 3 hematoxylin.

ALT and AST levels were analyzed in a routine laboratory with an automatic analyzer (Vitros 5.1, Ortho, France).

Genotyping of the *FAH* ^−/−^-iPS cells and *FAH* ^−/−^-iPS cell–derived mice was performed on genomic DNA isolated from the *FAH* ^−/−^-iPS cells, OG2 ES cells, and murine tail tips as reported previously [34].

### Generation of a Lentiviral Gene–Correction Construct

The self-inactivating (SIN) lentiviral vector pRRL.PPT.SFFV.FAH.2A.EGFP.Pre* was created based on pRRL.PPT.SFFV.EGFP.Pre* [64],[65]. Primers containing complementary restriction sites to the opened vector backbone as well as a Kozak consensus sequence were used to amplify murine *FAH* cDNA from plasmid *IRAVp968E0631D* (imaGenes, Berlin, Germany). The reverse primer deleted the stop codon of *FAH* cDNA to enable translation of a fusion protein in the final vector construct. The *EGFP* fragment of the lentiviral vector pRRL.PPT.SFFV.EGFP.Pre* plasmid was replaced with *FAH* cDNA. This vector pRRL.PPT.SFFV.FAH.Pre* was opened by restriction digestion for a second cloning step. Primers containing restriction sites as well as the sequence of the Porcine Teschovirus 2A oligopeptide [66] were used to amplify a *2A-EGFP* sequence and cloned into the opened vector backbone. The final vector construct contains a self-inactivating lentiviral vector expressing a fusion mRNA of *FAH* and *EGFP*. 2A peptide results in the cotranslational “cleavage” of the fusion protein and is an alternative to the internal ribosomal entry site. 2A peptides were shown to lead to expression of multiple cistrons at equimolar levels [67]. The vector pRRL.PPT.TTR.FAH.2A.EGFP.Pre* was created by replacing the promoter sequence from pRRL.PPT.SFFV.FAH.2A.EGFP.Pre* with the mini-*transthyretin* (*TTR*) promoter kindly provided by Dr. Weidong Xiao, Temple University, PA (PMID: 18500941). *FAH* expression was validated by RT-PCR and Western blot. Cloning details, vector maps, and sequence files are available upon request.

### Gene Correction of *FAH* ^−/−^-iPS Cells

Single-cell suspension (10,000 *FAH* ^−/−^ iPS cells, passage 7) was transduced with infectious particles of pRRL.PPT.SFFV.FAH.2A.EGFP.Pre* or pRRL.PPT.TTR.FAH.2A.EGFP.Pre* lentivirus (MOI = 100) in a volume of 50 µl (mESC medium supplemented with 10 µg ml^−1^ protamine sulfate [Sigma] and double concentration of LIF), and covered with mineral oil (Sigma). Following incubation for 8 h at 37°C in a humidified environment at 5% CO2, the cell suspension was transferred into a 6-cm dish with fresh MEFs and mouse ES cell medium.

## Supporting Information

Figure S1 Characterization of FAH^−/−^-iPS cells. (a) Specific PCR primers discriminating the FAH-knockout (KO) allele from the wild-type (Wt) allele confirmed the FAH^−/−^ genotype in FAH^−/−^-iPS cells. A weak Wt band was detected in the second lane, which was due to contamination with feeder cells (MEFs). This band was eliminated by manually picking the iPS cell colonies prior to DNA isolation (the third lane). (b) Efficient silencing of gamma retrovirus–mediated expression of the reprogramming factors in FAH^−/−^-iPS cells. Expression levels in freshly transduced cells (day 3) were set to 1.
(TIF)

Figure S2 Tetraploid embryo complementation using FAH^−/−^-iPS cells. (a) Two-cell embryos were flushed from 2.5-dpc mice. Scale bar, 100 µm. (b) To produce 4n embryos, 2-cell embryos were fused using electrofusion. Scale bar, 100 µm. (c) Compacting 4-cell embryos were observed 24 h after fusion. Scale bar, 100 µm. (d) The zonae pellucidae of the 4-cell embryos were removed by acidified Tyrode's solution and aggregated in pair with FAH^−/−^-iPS cells. Scale bar, 50 µm. (e) The chimeric embryos developed to the blastocyst stage in culture overnight and were then transferred into the uterus of pseudopregnant mice. Scale bar, 50 µm. (f) FAH^−/−^-iPS mice with their CD1 foster mother. Note the complete black fur color of these mice, in contrast to the brown fur color of the 4n host embryos. (g) Genotyping of the FAH^−/−^ allele (KO) and the wild-type allele (Wt) in pups that were derived from FAH^−/−^-iPS cells. Eight of 12 pups were shown to be entirely derived from FAH^−/−^-iPS cells. Four of the pups showed very mild contribution from the residual Wt cells of the 4n host embryos. (h) Genotyping of the FAH^−/−^ allele (KO) and the wild-type allele (Wt) in the F1 offspring resulting from the mating of FAH^−/−^-iPS cell–derived male mice with wild-type female mice.
(TIF)

Table S1 Copy numbers per genome for each reprogramming factor in the FAH^−/−^-iPS cells.
(TIF)

## Abbreviations

ALT: alanine aminotransferase
AST: aspartate aminotransferase
CGH: comparative genome hybridization
CNAs: copy number alterations
dpc: days post-coitum
FAH: fumarylacetoacetate hydrolase
H&E: hematoxylin and eosin
iPS: induced pluripotent stem
MEF: mouse embryonic fibroblast
qRT-PCR: quantitative real-time–PCR
SFFV: spleen focus-forming virus
SKY: spectral karyotyping
TTR: transthyretin

## References

[1] Takahashi K, Tanabe K, Ohnuki M, Narita M, Ichisaka T (2007). Induction of pluripotent stem cells from adult human fibroblasts by defined factors.. Cell.

[2] Takahashi K, Yamanaka S (2006). Induction of pluripotent stem cells from mouse embryonic and adult fibroblast cultures by defined factors.. Cell.

[3] Wernig M, Meissner A, Foreman R, Brambrink T, Ku M (2007). In vitro reprogramming of fibroblasts into a pluripotent ES-cell-like state.. Nature.

[4] Yu J, Vodyanik M. A, Smuga-Otto K, Antosiewicz-Bourget J, Frane J. L (2007). Induced pluripotent stem cell lines derived from human somatic cells.. Science.

[5] Hanna J, Wernig M, Markoulaki S, Sun C. W, Meissner A (2007). Treatment of sickle cell anemia mouse model with iPS cells generated from autologous skin.. Science.

[6] Park I. H, Arora N, Huo H, Maherali N, Ahfeldt T (2008). Disease-specific induced pluripotent stem cells.. Cell.

[7] Ye Z. H, Zhan H. C, Mali P, Dowey S, Williams D. M (2009). Human-induced pluripotent stem cells from blood cells of healthy donors and patients with acquired blood disorders.. Blood.

[8] Wang Y. X, Jiang Y. H, Liu S, Sun X. F, Gao S. R (2009). Generation of induced pluripotent stem cells from human beta-thalassemia fibroblast cells.. Cell Research.

[9] Dimos J. T, Rodolfa K. T, Niakan K. K, Weisenthal L. M, Mitsumoto H (2008). Induced pluripotent stem cells generated from patients with ALS can be differentiated into motor neurons.. Science.

[10] Ebert A. D, Yu J, Rose F. F, Mattis V. B, Lorson C. L (2009). Induced pluripotent stem cells from a spinal muscular atrophy patient.. Nature.

[11] Soldner F, Hockemeyer D, Beard C, Gao Q, Bell G. W (2009). Parkinson's disease patient-derived induced pluripotent stem cells free of viral reprogramming factors.. Cell.

[12] Lee G, Papapetrou E. P, Kim H, Chambers S. M, Tomishima M. J (2009). Modelling pathogenesis and treatment of familial dysautonomia using patient-specific iPSCs.. Nature.

[13] Maehr R, Chen S. B, Snitow M, Ludwig T, Yagasaki L (2009). Generation of pluripotent stem cells from patients with type 1 diabetes.. Proc Natl Acad Sci U S A.

[14] Moretti A, Bellin M, Welling A, Jung C. B, Lam J. T (2010). Patient-specific induced pluripotent stem-cell models for long-QT syndrome.. N Engl J Med.

[15] Carvajal-Vergara X, Sevilla A, D'Souza S. L, Ang Y. S, Schaniel C (2010). Patient-specific induced pluripotent stem-cell-derived models of LEOPARD syndrome.. Nature.

[16] Jin Z. B, Okamoto S, Osakada F, Homma K, Assawachananont J (2011). Modeling retinal degeneration using patient-specific induced pluripotent stem cells.. PLoS ONE.

[17] Raya A, Rodriguez-Piza I, Guenechea G, Vassena R, Navarro S (2009). Disease-corrected haematopoietic progenitors from Fanconi anaemia induced pluripotent stem cells.. Nature.

[18] Si-Tayeb K, Noto F. K, Nagaoka M, Li J, Battle M. A (2010). Highly efficient generation of human hepatocyte-like cells from induced pluripotent stem cells.. Hepatology.

[19] Sullivan G. J, Hay D. C, Park I. H, Fletcher J, Hannoun Z (2010). Generation of functional human hepatic endoderm from human induced pluripotent stem cells.. Hepatology.

[20] Hargus G, Cooper O, Deleidi M, Levy A, Lee K (2010). Differentiated Parkinson patient-derived induced pluripotent stem cells grow in the adult rodent brain and reduce motor asymmetry in Parkinsonian rats.. Proc Natl Acad Sci U S A.

[21] Polo J. M, Liu S, Figueroa M. E, Kulalert W, Eminli S (2010). Cell type of origin influences the molecular and functional properties of mouse induced pluripotent stem cells.. Nat Biotechnol.

[22] Kim K, Doi A, Wen B, Ng K, Zhao R (2010). Epigenetic memory in induced pluripotent stem cells.. Nature.

[23] Mayshar Y, Ben-David U, Lavon N, Biancotti J. C, Yakir B (2010). Identification and classification of chromosomal aberrations in human induced pluripotent stem cells.. Cell Stem Cell.

[24] Hussein S. M, Batada N. N, Vuoristo S, Ching R. W, Autio R (2011). Copy number variation and selection during reprogramming to pluripotency.. Nature.

[25] Gore A, Li Z, Fung H. L, Young J. E, Agarwal S (2011). Somatic coding mutations in human induced pluripotent stem cells.. Nature.

[26] Lister R, Pelizzola M, Kida Y. S, Hawkins R. D, Nery J. R (2011). Hotspots of aberrant epigenomic reprogramming in human induced pluripotent stem cells.. Nature.

[27] Wang Z. Q, Kiefer F, Urbanek P, Wagner E. F (1997). Generation of completely embryonic stem cell-derived mutant mice using tetraploid blastocyst injection.. Mech Dev.

[28] Nagy A, Rossant J, Nagy R, Abramow-Newerly W, Roder J. C (1993). Derivation of completely cell culture-derived mice from early-passage embryonic stem cells.. Proc Natl Acad Sci U S A.

[29] Nagy A, Gocza E, Diaz E. M, Prideaux V. R, Ivanyi E (1990). Embryonic stem cells alone are able to support fetal development in the mouse.. Development.

[30] Boland M. J, Hazen J. L, Nazor K. L, Rodriguez A. R, Gifford W (2009). Adult mice generated from induced pluripotent stem cells.. Nature.

[31] Kang L, Wang J, Zhang Y, Kou Z, Gao S (2009). iPS cells can support full-term development of tetraploid blastocyst-complemented embryos.. Cell Stem Cell.

[32] Zhao X. Y, Li W, Lv Z, Liu L, Tong M (2009). iPS cells produce viable mice through tetraploid complementation.. Nature.

[33] Stadtfeld M, Apostolou E, Akutsu H, Fukuda A, Follett P (2010). Aberrant silencing of imprinted genes on chromosome 12qF1 in mouse induced pluripotent stem cells.. Nature.

[34] Grompe M, al-Dhalimy M, Finegold M, Ou C. N, Burlingame T (1993). Loss of fumarylacetoacetate hydrolase is responsible for the neonatal hepatic dysfunction phenotype of lethal albino mice.. Genes Dev.

[35] Meaking W. S, Edgerton J, Wharton C. W, Meldrum R. A (1995). Electroporation-induced damage in mammalian cell DNA.. Biochim Biophys Acta.

[36] Lakshmipathy U, Buckley S, Verfaillie C (2007). Gene transfer via nucleofection into adult and embryonic stem cells.. Methods Mol Biol.

[37] Zou J, Maeder M. L, Mali P, Pruett-Miller S. M, Thibodeau-Beganny S (2009). Gene targeting of a disease-related gene in human induced pluripotent stem and embryonic stem cells.. Cell Stem Cell.

[38] Montini E, Cesana D, Schmidt M, Sanvito F, Bartholomae C. C (2009). The genotoxic potential of retroviral vectors is strongly modulated by vector design and integration site selection in a mouse model of HSC gene therapy.. J Clin Invest.

[39] Modlich U, Navarro S, Zychlinski D, Maetzig T, Knoess S (2009). Insertional transformation of hematopoietic cells by self-inactivating lentiviral and gammaretroviral vectors.. Mol Ther.

[40] Schambach A, Baum C (2008). Clinical application of lentiviral vectors - concepts and practice.. Curr Gene Ther.

[41] Cartier N, Hacein-Bey-Abina S, Bartholomae C. C, Veres G, Schmidt M (2009). Hematopoietic stem cell gene therapy with a lentiviral vector in X-linked adrenoleukodystrophy.. Science.

[42] Aiuti A, Ferrua F, Scaramuzza S, Bosticardo M, Castiello C (2010). Gene therapy trial with lentiviral vector transduced CD34+cells for the treatment of Wiskott-Aldrich Syndrome.. Human Gene Therapy.

[43] Espejel S, Roll G. R, McLaughlin K. J, Lee A. Y, Zhang J. Y (2010). Induced pluripotent stem cell-derived hepatocytes have the functional and proliferative capabilities needed for liver regeneration in mice.. J Clin Invest.

[44] Rudolph C, Schlegelberger B (2009). Spectral karyotyping and fluorescence in situ hybridization of murine cells.. Methods Mol Biol.

[45] Grompe M, Lindstedt S, al-Dhalimy M, Kennaway N. G, Papaconstantinou J (1995). Pharmacological correction of neonatal lethal hepatic dysfunction in a murine model of hereditary tyrosinaemia type I.. Nat Genet.

[46] Ohta H, Sakaide Y, Yamagata K, Wakayama T (2008). Increasing the cell number of host tetraploid embryos can improve the production of mice derived from embryonic stem cells.. Biol Reprod.

[47] Kim J. B, Zaehres H, Wu G, Gentile L, Ko K (2008). Pluripotent stem cells induced from adult neural stem cells by reprogramming with two factors.. Nature.

[48] Silva J, Barrandon O, Nichols J, Kawaguchi J, Theunissen T. W (2008). Promotion of reprogramming to ground state pluripotency by signal inhibition.. PLoS Biol.

[49] Kim J. B, Greber B, Arauzo-Bravo M. J, Meyer J, Park K. I (2009). Direct reprogramming of human neural stem cells by OCT4.. Nature.

[50] Kim J. B, Sebastiano V, Wu G, Arauzo-Bravo M. J, Sasse P (2009). Oct4-induced pluripotency in adult neural stem cells.. Cell.

[51] Kiskinis E, Eggan K (2010). Progress toward the clinical application of patient-specific pluripotent stem cells.. J Clin Invest.

[52] Wernig M, Lengner C. J, Hanna J, Lodato M. A, Steine E (2008). A drug-inducible transgenic system for direct reprogramming of multiple somatic cell types.. Nat Biotechnol.

[53] Overturf K, Al-Dhalimy M, Tanguay R, Brantly M, Ou C. N (1996). Hepatocytes corrected by gene therapy are selected in vivo in a murine model of hereditary tyrosinaemia type I.. Nat Genet.

[54] Montini E, Held P. K, Noll M, Morcinek N, Al-Dhalimy M (2002). In vivo correction of murine tyrosinemia type I by DNA-mediated transposition.. Mol Ther.

[55] Held P. K, Olivares E. C, Aguilar C. P, Finegold M, Calos M. P (2005). In vivo correction of murine hereditary tyrosinemia type I by phiC31 integrase-mediated gene delivery.. Mol Ther.

[56] Paulk N. K, Wursthorn K, Wang Z, Finegold M. J, Kay M. A (2009). Adeno-associated virus gene repair corrects a mouse model of hereditary tyrosinemia in vivo.. Hepatology.

[57] Overturf K, al-Dhalimy M, Ou C. N, Finegold M, Grompe M (1997). Serial transplantation reveals the stem-cell-like regenerative potential of adult mouse hepatocytes.. Am J Pathol.

[58] Azuma H, Paulk N, Ranade A, Dorrell C, Al-Dhalimy M (2007). Robust expansion of human hepatocytes in Fah-/-/Rag2-/-/Il2rg-/- mice.. Nat Biotechnol.

[59] Sharma A. D, Cantz T, Vogel A, Schambach A, Haridass D (2008). Murine embryonic stem cell-derived hepatic progenitor cells engraft in recipient livers with limited capacity of liver tissue formation.. Cell Transplant.

[60] Szabo P. E, Hubner K, Schöler H, Mann J. R (2002). Allele-specific expression of imprinted genes in mouse migratory primordial germ cells.. Mech Dev.

[61] Cantz T, Bleidissel M, Stehling M, Schöler H. R (2008). In vitro differentiation of reprogrammed murine somatic cells into hepatic precursor cells.. Biol Chem.

[62] Gaubatz J. W, Cutler R. G (1978). Age-related differences in the number of ribosomal RNA genes of mouse tissues.. Gerontology.

[63] Zhou H, Wu S, Joo J. Y, Zhu S, Han D. W (2009). Generation of induced pluripotent stem cells using recombinant proteins.. Cell Stem Cell.

[64] Schambach A, Galla M, Modlich U, Will E, Chandra S (2006). Lentiviral vectors pseudotyped with murine ecotropic envelope: increased biosafety and convenience in preclinical research.. Exp Hematol.

[65] Schambach A, Bohne J, Baum C, Hermann F. G, Egerer L (2006). Woodchuck hepatitis virus post-transcriptional regulatory element deleted from X protein and promoter sequences enhances retroviral vector titer and expression.. Gene Ther.

[66] Szymczak A. L, Vignali D. A (2005). Development of 2A peptide-based strategies in the design of multicistronic vectors.. Expert Opin Biol Ther.

[67] Torres V, Barra L, Garces F, Ordenes K, Leal-Ortiz S (2010). A bicistronic lentiviral vector based on the 1D/2A sequence of foot-and-mouth disease virus expresses proteins stoichiometrically.. J Biotechnol.

