# Supplementary figures and images for: Generation of Healthy Mice from Gene-Corrected Disease-Specific Induced Pluripotent Stem Cells
Source: PLoS Biol. 2011 Jul 12;9(7):e1001099. doi: 10.1371/journal.pbio.1001099 (PMC3134447; doi:10.1371/journal.pbio.1001099)

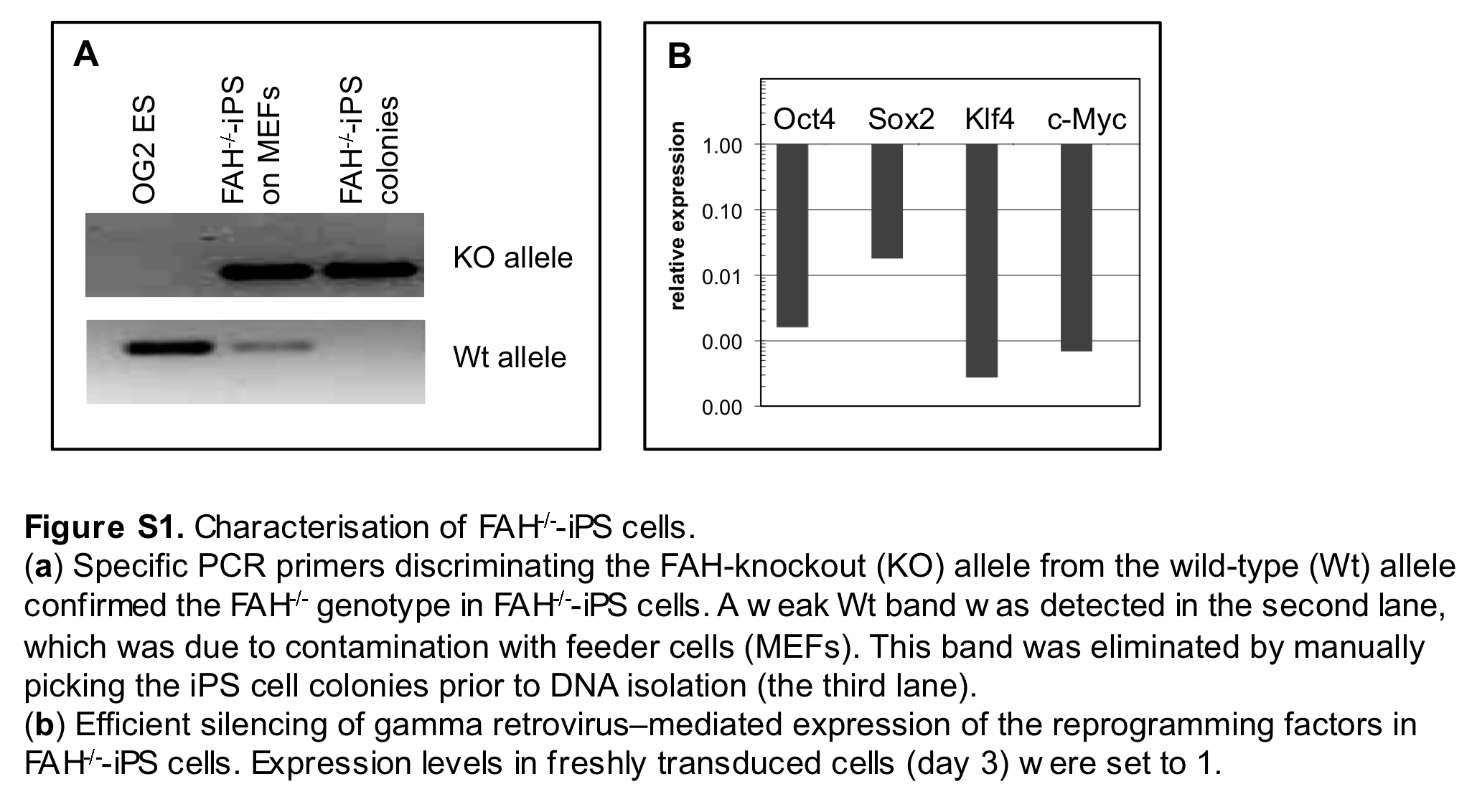

Supplement: Figure S1 — Characterization of FAH−/−-iPS cells. (a) Specific PCR primers discriminating the FAH-knockout (KO) allele from the wild-type (Wt) allele confirmed the FAH−/− genotype in FAH−/−-iPS cells. A weak Wt band was detected in the second lane, which was due to contamination with feeder cells (MEFs). This band was eliminated by manually picking the iPS cell colonies prior to DNA isolation (the third lane). (b) Efficient silencing of gamma retrovirus–mediated expression of the reprogramming factors in FAH−/−-iPS cells. Expression levels in freshly transduced cells (day 3) were set to 1. (TIF) [file pbio.1001099.s001.tif]

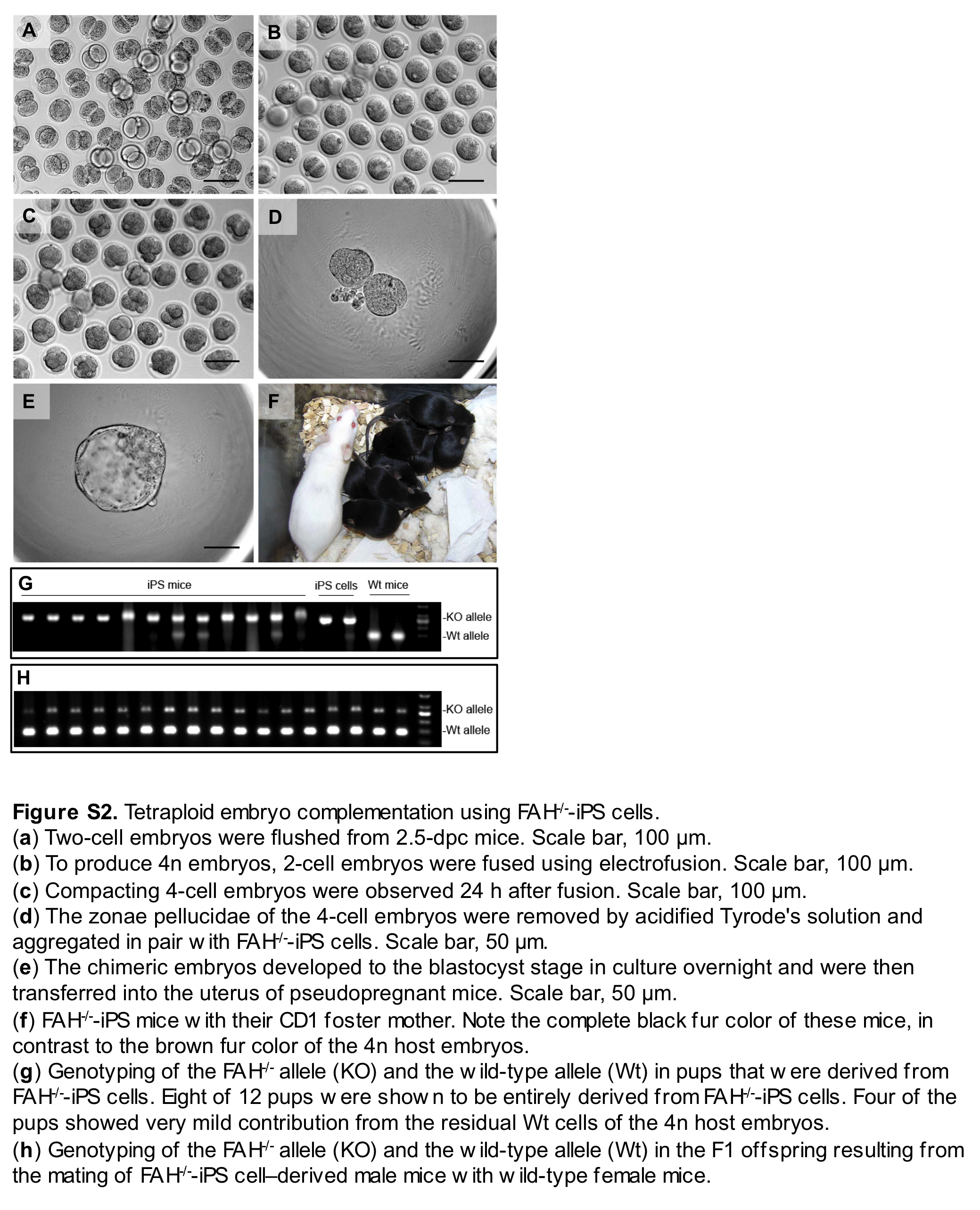

Supplement: Figure S2 — Tetraploid embryo complementation using FAH−/−-iPS cells. (a) Two-cell embryos were flushed from 2.5-dpc mice. Scale bar, 100 µm. (b) To produce 4n embryos, 2-cell embryos were fused using electrofusion. Scale bar, 100 µm. (c) Compacting 4-cell embryos were observed 24 h after fusion. Scale bar, 100 µm. (d) The zonae pellucidae of the 4-cell embryos were removed by acidified Tyrode's solution and aggregated in pair with FAH−/−-iPS cells. Scale bar, 50 µm. (e) The chimeric embryos developed to the blastocyst stage in culture overnight and were then transferred into the uterus of pseudopregnant mice. Scale bar, 50 µm. (f) FAH−/−-iPS mice with their CD1 foster mother. Note the complete black fur color of these mice, in contrast to the brown fur color of the 4n host embryos. (g) Genotyping of the FAH−/− allele (KO) and the wild-type allele (Wt) in pups that were derived from FAH−/−-iPS cells. Eight of 12 pups were shown to be entirely derived from FAH−/−-iPS cells. Four of the pups showed very mild contribution from the residual Wt cells of the 4n host embryos. (h) Genotyping of the FAH−/− allele (KO) and the wild-type allele (Wt) in the F1 offspring resulting from the mating of FAH−/−-iPS cell–derived male mice with wild-type female mice. (TIF) [file pbio.1001099.s002.tif]

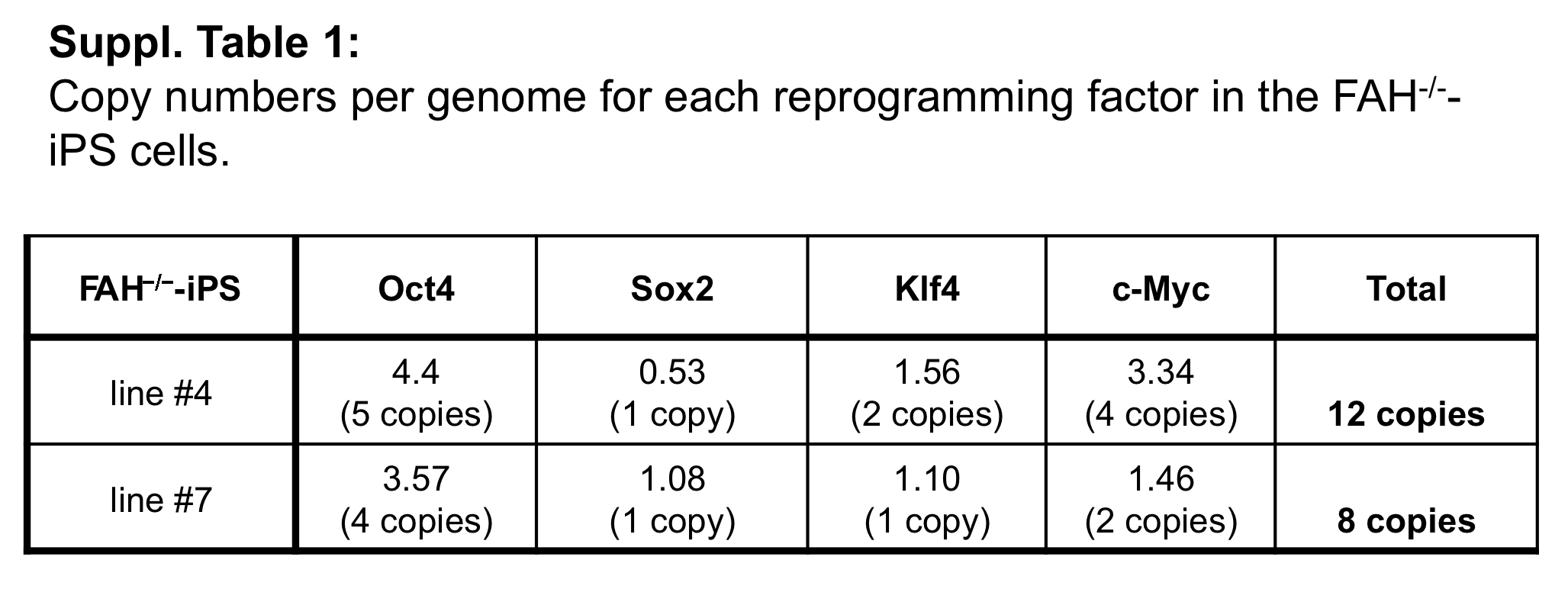

Supplement: Table S1 — Copy numbers per genome for each reprogramming factor in the FAH−/−-iPS cells. (TIF) [file pbio.1001099.s003.tif]
